# Facilitators and barriers for healthcare providers to recommend HPV vaccination to attendees of public sexually transmitted diseases clinics in Hong Kong, China

**DOI:** 10.1371/journal.pone.0209942

**Published:** 2019-01-09

**Authors:** Ying Ying Lee, Zixin Wang

**Affiliations:** 1 Department of Health, Hong Kong SAR, China; 2 Centre for Health Behaviours Research, JC School of Public Health and Primary Care, The Chinese University of Hong Kong, Hong Kong SAR, China; University of Campania, ITALY

## Abstract

**Background:**

Attendees of sexually transmitted diseases (STD) clinics could also benefit from HPV vaccination. Healthcare providers’ recommendation is the most effective published method in motivating HPV vaccination initiation. This study was to investigate practice of recommending HPV vaccination to attendees among healthcare providers (doctors and nurses) working in public STD clinics in Hong Kong, China.

**Method:**

Participants were medical doctors, registered nurses and enrolled nurses working in all eight public STD clinics in Hong Kong. All of them (29 doctors and 82 nurses) were approached by telephone. A self-administered questionnaire was mailed to them. A total of 98 completed questionnaires were received (28 doctors and 70 nurses). The study was conducted during January to May, 2018. Using recommendation of HPV vaccination to any attendees in the last year as the dependent variable, univariate and multiple logistic regression models were fitted.

**Results:**

In the last 12 months, 16.3% and 36.7% of the participants had recommended HPV vaccination to any male attendees and to any female attendees of their clinics; 41.8% had recommended it to either male or female attendees. Adjusting for significant background variables (professions and years of working experience in the clinic), three constructs of the Theory of Planned Behavior were significantly associated with the dependent variable in expected directions. They were: 1) the Positive Attitude Scale (adjusted odds ratios, AOR: 1.20, 95%CI: 1.02, 1.41), 2) the Negative Attitude Scale (AOR: 0.85, 95%CI: 0.77, 0.94), and 3) the Perceived Behavioral Control Scale (AOR: 1.37, 95%CI: 1.08, 1.75).

**Conclusion:**

STD clinics are ideal settings that allow healthcare providers to access individuals who are at high-risk of HPV infection and promote HPV vaccination. Health promotion targeting these healthcare providers is warranted to enhance their perceived importance of recommending HPV vaccination. Such promotion should modify their attitudes and perceived behavioral control related to recommending HPV vaccination to attendees.

## Introduction

Human papillomavirus (HPV) vaccination is highly effective in preventing vaccine-type general warts and cancers among both males and females [1, 2]. Growing evidences suggested that sexually active men and women could also benefit from HPV vaccination. People who have already infected with one or more HPV types can still get protection from other HPV types in the vaccines [3]. HPV vaccination can also significantly reduce the risk of developing/recurrence of subsequent HPV-related diseases (e.g., cervical/anal intraepithelial neoplasia) among people having history of HPV infection [4–6]. Given such development, the U.S. Centers for Disease Control and Prevention (CDC) recommends HPV vaccination for young men (≤21 years) and women (≤26 years), as well as some high-risk groups (e.g., gay, bisexual, transgender people, and people living with HIV age ≤26 years) who had not been vaccinated previously [3].

Bivalent, 4-valent and 9-valent HPV vaccines are available in Hong Kong where the study was conducted. These vaccines are provided by private physicians to males and females age ≥9 years. The cost to complete the 3-dose course ranges from HK$2,500 to 4,000 (US$ 321–514) [7]. There are eight public sexually transmitted diseases (STD) clinics in Hong Kong. They are main service providers for STD management, prevention and control. The number of attendees of these clinics was 12,325 in 2016 [8], many of them are having high risk behaviors and hence at high risk of contracting HPV and HPV-related diseases. In 2017, 2,007 new cases of genital warts were diagnosed by these clinics, and such number has been increasing overtime [9]. STD clinic attendees may benefit from HPV vaccination [4–6]. Given the very low uptake of HPV vaccination among males (0–0.6%) [10, 11] and females (2.2–9.1%) [10, 12] in Hong Kong, the uptake rate is estimated to be very low among STD clinic attendees. Promoting HPV vaccination among STD clinic attendees should be considered.

Healthcare providers’ recommendation is regarded as the most effective method in motivating HPV vaccination initiation [13–16]. Thirteen quantitative studies and one qualitative study examined attitudes and practice related to recommending HPV vaccination to patients/clients among healthcare providers in various countries [17–29]. Understanding healthcare providers’ facilitators and barriers of recommending HPV vaccination is important for developing effective intervention. Higher knowledge of HPV vaccination and perceived HPV vaccination to be effective in preventing HPV-related diseases for their patients/clients were found to be facilitators [20, 23, 30]. Barriers to recommend HPV vaccination included lack of guideline or protocol for recommending HPV vaccination to patients/clients, concerns that they would be regarded as selling an expensive vaccine or labelling their patients/clients, and worry that their patients/clients would be reluctant to discuss sexuality and STD [20, 22, 23, 25]. These factors were considered by this study. A meta-analysis showed that theory-based interventions are more effective than non-theory-based [31]. The Theory of Planned Behavior (TPB) [32] is a commonly used theory which postulates that in order to adopt a health-related behavior (e.g., recommend HPV vaccination), one would first evaluate the behavior (positive and negative attitudes), consider whether their significant others would support such behavior (perceived subjective norm), and appraise how much control they have over the behavior (perceived behavioral control) [32]. We used TPB as the framework of this study and tested significance of its constructs.

In this study, we surveyed doctors and nurses working in all eight public STD clinics in Hong Kong, China. Their practices of recommending HPV vaccination to the clinic attendees in the last year were investigated. Associated factors were also investigated. These factors included their background characteristics, knowledge related to HPV vaccination and perceptions related to recommending HPV vaccination based on the TPB.

## Method

### Study design and participants

An anonymous cross-sectional survey was conducted among healthcare providers working in public STD clinics in Hong Kong during January to May, 2018. Participants were medical doctors, registered nurses and enrolled nurses working in public STD clinics in Hong Kong. There are 29 doctors and 82 nurses currently working in eight public STD clinics in Hong Kong.

### Pilot study

The questionnaire was piloted among three healthcare providers (one doctor, one registered nurse, and one enrolled nurse) working in public STD clinics. Based on the feedback, the questionnaire was modified and finalized by the authors (S1 Survey questionnaire). Data obtained from these three healthcare providers was not included in the data analysis.

### Data collection

Through telephone, the first author approached all medical doctors and nursing officers in these clinics, briefed them about the study, and invited them to participate. She also invited the nursing officers to pass the study information and invitation to all their nursing colleagues. Oral informed consent was obtained before a self-administered questionnaire was mailed to each prospective participant. A statement was printed on the questionnaire indicating that completion of the survey implied consent to join this study. Return envelops with stamps were enclosed for participants to send back the completed questionnaires. No incentive was given to the participants. A total of 98 completed questionnaires were received (28 doctors and 70 nurses). Ethics approvals were obtained from both the Survey and Behavioral Research Ethics Committee of the Chinese University of Hong Kong and the Department of Health, Hong Kong SAR.

### Measures

#### Socio-demographic variables

Information collected included professions, gender, age, marital status, religion, education level and years of working experience in STD clinics.

#### Recommendation of HPV vaccination to STD clinic attendees

Participants were asked whether they had recommended HPV vaccination to any male and female attendees of their clinics in the last 12 months. A composite variable was created representing recommendation of HPV vaccination to any attendees (either males or females) in the last 12 months. This composite variable was used the dependent variable in subsequent logistic regression analysis. Number of male and female attendees that they have recommended HPV vaccination to was also recorded.

#### Knowledge related to HPV vaccination

Seven items were used to assess knowledge related to HPV vaccination (e.g., ‘People who are sexually active cannot receive HPV vaccination’). A composite variable was constructed by counting the number of correct responses to knowledge items with the range from 0 to 7.

#### Perceptions related to recommending HPV vaccination to STD clinic attendees

Four scales were constructed to assess participants’ perceptions related to recommending HPV vaccination. They were based on the TPB.

Positive attitudes toward recommending HPV vaccination to STD clinic attendees were measured by four items (e.g., ‘HPV vaccination is highly effective in preventing HPV/HPV-related diseases among sexually active men and women’). The Positive Attitude Scale was formed by summing up individual item scores. Higher score on the scale indicated more positive attitude towards recommending HPV vaccination to attendees of their clinics.

Negative attitudes toward recommending HPV vaccination to STD clinic attendees were measured by seven items (e.g., ‘recommending HPV vaccination to attendees of your clinic would be regarded as hard selling an expensive vaccine’). The Negative Attitude Scale was formed by summing up individual item scores. Higher score on the scale indicated more negative attitude towards recommending HPV vaccination to attendees of their clinics.

Two items were used to measure participants’ perceived support from their significant others (their colleagues and supervisors) for recommending HPV vaccination to STD clinic attendees. The Subjective Norm Scale was formed by summing up individual item scores. Higher score on the scale indicated perceived subjective norm more supportive of giving such recommendations.

Another three items were used to measure perceived behavioral control in recommending HPV vaccination to STD clinic attendees (e.g., ‘I am confident that I can recommend HPV vaccine to STD clinic attendees, if there is guideline/protocol from the department’). The Perceived Behavioral Control Scale was formed by summing up individual item scores. Higher score on the scale indicated higher behavioral control in giving such recommendations.

The response categories of all items ranged from 1 (strongly disagree) to 5 (strongly agree). Cronbach’s alpha of these four scales ranged from 0.57 to 0.89.

### Statistical analysis

Descriptive statistics of all studied variables were presented. Using recommendation of HPV vaccination to any attendees of their clinics (either males or female) in the last 12 months as the dependent variable, univariate odds ratios (ORu) for the associations between background independent variables and the dependent variable were estimated. Those background variables with p<0.10 in the univariate analysis were adjusted for the subsequent multiple logistic regression analysis. Three multiple logistic regression models were constructed in this study. Each model contained three variables, they were: 1) years of working experiences in the STD clinic, 2) professions, and 3) one scale measuring perceptions based on the TPB (the Positive Attitude Scale, the Negative Attitude Scale, or the Perceived Behavioral Control Scale). Adjusted odds ratios (AOR) and respective 95% confidence interval (CI) were derived from such analyses. SPSS version 24.0 was used for data analysis, with p values <0.05 taken as statistically significant.

## Results

### Socio-demographic variables

Over half of the participants were aged >40 years (53.1%), female (52.0%), married (80.6%), had obtained bachelor degree or above (83.7%), and without any religion (70.4%). Majority of them had at least 5 years’ working experience in STD clinics (56.1%), and self-reported seeing at least 100 attendees per week in their STD clinics (70.4%) (Table 1). There was no difference in aforementioned background characteristics between respondents and non-respondents among nurses (data not tabulated).

**Table 1:** Background characteristics of participants (n = 98).

	n	%
Age group		
20–40	46	46.9
41–50	21	21.4
>50	31	31.6
Gender		
Male	47	48.0
Female	51	52.0
Marital status		
Currently single	19	19.4
Married	79	80.6
Education level		
Diploma	16	16.3
Bachelor degree	44	44.9
Postgraduate diploma and above	38	38.8
Religion		
No	69	70.4
Yes	29	29.6
Professions		
Doctor	28	28.6
Registered nurse	64	67.3
Enrolled nurse	6	6.1
Years of working experience in the STD clinic		
<5 years	43	43.9
5–10 years	20	20.4
> 10 years	35	35.7
Number of attendees seen per week in the STD clinic		
<100	29	29.6
≥100	69	70.4

STD: sexually transmitted diseases

### Recommendation of HPV vaccination to attendees of STD clinics

In the last 12 months, 16.3% and 36.7% of the participants had recommended HPV vaccination to any male attendees and to any female attendees of their clinics; 41.8% had recommended it to either male or female attendees. Among those who had given such recommendation, 6.2% and 22.2% had recommended to over 10 male and female attendees, respectively (Table 2).

**Table 2:** Knowledge and perceptions related to HPV vaccination (n = 98).

	%	Mean (SD)
**Recommendation of HPV vaccination to attendees of STD clinics in the last 12 months**		
Recommendation of HPV vaccination to any male STD clinic attendees		
No	83.7	
Yes	16.3	
Recommendation of HPV vaccination to any female STD clinic attendees		
No	63.3	
Yes	36.7	
Recommendation of HPV vaccination to any STD clinic attendees (either male or female)		
No	58.2	
Yes	41.8	
Number of male STD clinic attendees they had recommended HPV vaccination to (among those who had recommended HPV vaccination to any male STD clinic attendees, n = 16)		
<5	75.0	
5–10	18.8	
>10	6.2	
Number of female STD clinic attendees they had recommended HPV vaccination to (among those who had recommended HPV vaccination to any female STD clinic attendees, n = 36)		
<5	52.8	
5–10	25.0	
>10	22.2	
**Knowledge related to HPV vaccination**		
Is there a vaccine that protects against HPV for women		
Yes*	94.9	
No/not sure	5.1	
Is there a vaccine that protects against HPV for men		
Yes*	76.5	
No/not sure	23.5	
People who are sexually active cannot receive HPV vaccination		
Yes	1.0	
No*	99.0	
People who have been diagnosed with HPV cannot be given HPV vaccination		
Yes	8.2	
No*	91.8	
People with genital warts cannot be given HPV vaccination		
Yes	6.1	
No*	93.9	
HIV-positive people cannot get HPV vaccination		
Yes	8.2	
No*	91.8	
HPV vaccination is safe for pregnant women		
Yes	29.6	
No*	70.4	
Number of correct responses to HPV-related knowledge		6.2 (0.9)
**Perceptions related to recommending HPV vaccination to STD clinic attendees**		
Positive attitudes toward recommending HPV vaccination to STD clinic attendees (% agree/strongly agree)		
HPV vaccination is highly effective in preventing HPV/HPV-related diseases among sexually active men and women	67.3	
People who have already infected with one or more HPV types can still get protection from other HPV types in the vaccines	37.8	
For people who are infected with HPV, HPV vaccination is effective in protecting them from having genital warts or related cancers	27.6	
HPV vaccine is highly effective in preventing HPV/HPV-related diseases among HIV positive people	43.9	
*Positive Attitude Scale* ^1^		12.9 (2.9)
Negative attitudes toward recommending HPV vaccination to STD clinic attendees (% agree/strongly agree)		
Recommending HPV vaccines to patients will be regarded as hard selling an expensive vaccine	17.3	
Time is not sufficient in each consultation session for recommending HPV vaccines to patients	63.3	
It is difficult to initiate the conversation about HPV vaccines in the consultation	32.7	
HPV vaccination is not related to the reasons of consultation	40.8	
It is not my responsibility to recommend HPV vaccines to patients	26.5	
There is lack of guideline or protocol for me to recommend HPV vaccines to patients	72.4	
Recommendation of HPV vaccines will be given to patients only if vaccination is publicly funded	36.7	
*Negative Attitude Scale* ^2^		21.6 (5.2)
Subjective norm related to recommending HPV vaccination to STD clinic attendees (% agree/strongly agree)		
Your colleagues would support you to recommend HPV vaccination to attendees of your clinic	11.2	
Your supervisors would support you to recommend HPV vaccination to attendees of your clinic	7.1	
*Subjective Norm Scale* ^3^		5.0 (1.8)
Perceived behavioral control related to recommending HPV vaccination to STD clinic attendees (% agree/strongly agree)		
You are confident that you can recommend HPV vaccination to attendees of your clinic even the consultation work is busy	29.6	
You are confident that you can recommend HPV vaccination to attendees of your clinic if you have sufficient information about the vaccines	58.2	
You are confident that you can recommend HPV vaccine to HPV vaccination to attendees of your clinic if there is guideline / protocol from the department	73.5	
*Perceived Behavioral Control Scale* ^4^		10.4 (2.0)

STD: sexually transmitted diseases

* correct response to knowledge items

^1^ Positive Attitude Scale, 4 items, Cronbach alpha = 0.70

^2^ Negative Attitude Scale, 7 items, Cronbach alpha = 0.76

^3^ Subjective Norm Scale, 2 items, Cronbach alpha = 0.89

^4^ Perceived Behavioral Control Scale, 3 items, Cronbach alpha = 0.57

### Knowledge of HPV vaccination and perceptions related to recommendation of HPV vaccination to STD clinic attendees

The prevalence of correct response to seven knowledge items related to HPV vaccination ranged from 70.4% to 99.0%. Item responses and means (standard deviation, SD) of the scales measuring perceptions related to recommendation of HPV vaccination to STD clinic attendees were described in Table 2.

### Factors associated with recommendation of HPV vaccination to any STD clinic attendees

As compared to healthcare providers with <5 years working experiences, those having 5–10 years working experience were more likely to have recommended HPV vaccination to any STD clinic attendees in the last 12 months (70.0% versus 35.3%, ORu: 4.36, 95%CI: 1.39, 13.67, p = 0.012). The difference in prevalence of the dependent variable between doctors and registered nurses was of marginal statistical significant (55.6% versus 35.9%, ORu: 0.45, 95%CI: 0.18, 1.12, p = 0.086) (Table 3). Adjusting for these two background variables, three out of four constructs of the TPB were significantly associated with the dependent variable in expected directions. They were: 1) the Positive Attitude Scale (AOR: 1.20, 95%CI: 1.02, 1.41), 2) the Negative Attitude Scale (AOR: 0.85, 95%CI: 0.77, 0.94), and 3) the Perceived Behavioral Control Scale (AOR: 1.37, 95%CI: 1.08, 1.75) (Table 4).

**Table 3:** Associations between background variables and recommendation of HPV vaccination to any STD clinic attendees (either male or female) in the last 12 months (n = 98).

	%	Row%	ORu (95%CI)	P value
Age group				
20–40	46.9	50.0	1.0	
41–50	21.4	38.1	0.62 (0.22, 1.77)	0.366
>50	31.6	33.3	0.48 (0.18, 1.23)	0.154
Gender				
Male	48.0	34.8	1.0	
Female	52.0	49.0	1.86 (0.82, 4.21)	0.158
Marital status				
Currently single	19.4	36.8	1.0	
Married	80.6	43.6	1.30 (0.46, 3.64)	0.594
Education level				
Diploma	16.3	37.5	1.0	
Bachelor degree	44.9	45.5	1.39 (0.43, 4.49)	0.583
Postgraduate diploma and above	38.8	40.5	1.09 (0.33, 3.62)	0.835
Religion				
No	70.4	43.5	1.0	
Yes	29.6	39.3	0.79 (0.33, 1.93)	0.705
Professions				
Doctor	28.6	55.6	1.0	
Registered nurse	67.3	35.9	0.45 (0.18, 1.12)	0.086
Enrolled nurse	6.1	50.0	0.80 (0.14, 4.70)	0.805
Years of working experience in the STD clinic				
<5 years	43.9	35.3	1.0	
5–10 years	20.4	70.0	**4.36 (1.39, 13.67)**	**0.012**
> 10 years	35.7	34.9	0.97 (0.38, 2.49)	0.970
Number of attendees seen per week in the STD clinic				
<100	29.6	46.4	1.0	
≥100	70.4	40.6	0.84 (0.35, 2.02)	0.598

ORu: univariate odds ratios; CI: confidence interval

ORu, 95%CI and P values for variables with p<0.05 were bold

**Table 4:** Factors associated with recommendation of HPV vaccination to any STD clinic attendees (either male or female) in the last 12 months (n = 98).

	ORu (95%CI)	AOR (95%CI)
**Knowledge related to HPV vaccination**		
HPV-related knowledge score	1.48 (0.88, 2.46)	—
**Perceptions related to HPV vaccination**		
Positive Attitude Scale	1.20 (1.03, 1.40)*	1.20 (1.02, 1.41)*
Negative Attitude Scale	0.89 (0.81, 0.97)***	0.85 (0.77, 0.94)**
Subjective Norm Scale	1.04 (0.83, 1.30)	**—**
Perceived Behavioral Control Scale	1.32 (1.06, 1.65)**	1.37 (1.08, 1.75)*

ORu: univariate odds ratios; CI: confidence interval

ORu, AOR and 95%CI for variables with p<0.05 were bold

AOR: adjusted odds ratios, odds ratios adjusted for professions and years of working experiences.

* P < 0.05

** P < 0.01

*** P < 0.001

## Discussion

Embarrassment to talk about sex-related topics and perceived getting vaccinated for HPV might be seen as a symbol of promiscuous were common barriers for sexually active adults to take up HPV vaccination [11, 33]. STD clinics are ideal settings that allow healthcare providers to access groups that are of high risk of HPV infection. It may be easier to promote HPV vaccination among these high risk groups than in other settings, as it is natural for STD clinic attendees to receive counseling related to sexual health; attendees may feel less stigma and embarrassed. Interventions using setting approaches have been shown to be effective. Recommendation from healthcare providers of STD clinics may be a strong cue to action in motivating HPV vaccination initiation among STD clinic attendees. Previous studies consistently supported the pivotal role of healthcare providers in promoting vaccination against various types of diseases (e.g., HPV, seasonal influenza, etc.) in different settings and among different at-risk groups [34–36].

Relatively few healthcare providers working in the public STD clinics in Hong Kong have recommended HPV vaccination to attendees of their clinics (41.8%). The proportion of the participants who have recommended HPV vaccination to female attendees (36.7%) was lower than general physicians, obstetricians, gynecologists and pediatricians in other countries (47.0–83.8%) [18, 22, 25, 28]. HPV vaccination for males is a new initiative, which is expected to facilitate the prevention of HPV-related diseases in both genders. However, recent studies showed that HPV vaccination uptake was much lower among male adolescents/young men than their female counterparts [10, 11]. Many male adolescents and their parents were unaware of HPV vaccination for males, and hence showed low willingness to vaccinate male adolescents against HPV [10, 11, 37–39]. Similar situation applied to healthcare providers in our study, as only 16.7% of them have recommended HPV vaccination to male attendees. Such proportion was comparable to pediatricians in the U.S. and Italy (12.0–18.4%) [21, 28].

Since the number of healthcare providers in public STD clinics is relatively small, health promotion to improve the current situation should be feasible. The findings of this study provided some insights for developing such health promotion. As compared to doctors, registered nurses had lower likelihood of recommending HPV vaccination to attendees. It is understandable as nurses in Hong Kong are less involved in decision making related to diagnosis, prescription and treatment. Since nurses have the most contact with the patients, they can play important roles in future HPV vaccination promotion. Healthcare providers with longer working experience in STD clinics had higher likelihood of recommending HPV vaccination to attendees, as compared to those having less experience. Since HPV vaccines became available in Hong Kong since 2006, participants having longer working experience might have exposed to more information disseminated by pharmaceutical companies. However for those with more than 10 years’ experience, many of them are supervisors in these clinics. They have fewer number of consultation and hence less chance of giving recommendation to attendees. More attention should be given to registered nurses and healthcare providers with less working experience in the STD clinics in future health promotion.

Our participants had good knowledge about HPV vaccination for females, as nearly 95% of them knew that HPV vaccination could protect females. Such proportion was comparable to healthcare providers in Italy [28], and was higher than those in India and Norway [18, 27]. However, fewer of them (76.5%) knew there was a vaccine that protected against HPV for males. Such proportion was lower than healthcare providers in some western countries [28]. Contrary to expectations, future promotion enhancing knowledge about HPV vaccination may not be useful in motivating healthcare providers to recommend HPV vaccination to attendees, as it was a non-significant factor.

The TPB is a potential useful framework to guide the design of future health promotion targeting healthcare providers in STD clinics, as three out of four constructs of TPB were significantly associated with recommendation of HPV vaccination in expected directions. It is important to strengthen positive attitudes toward recommending HPV vaccination. Although over two-third of the healthcare providers perceived that HPV vaccination was effective in preventing HPV/HPV-related diseases among sexually active people in general, fewer of them (27.6–43.9%) believed that similar efficacies applied to those with history of HPV and/or HIV infection. Since HPV vaccination research is a rapid developing area, healthcare providers should be regularly updated about the new evidences in this area. Future training for healthcare providers should be conducted. The contents of the training should focus on the fact that both sexually active men and women who have already infected with one or more HPV types could benefit from HPV vaccination.

It is also important to remove some negative attitudes, as they were negatively associated with recommendation of HPV vaccination to STD clinic attendees. Over 70% of them indicated that there was lack of guideline or protocol for them to recommend HPV vaccination to attendees, and 26.5% hence perceived it was not their responsibility to do so. Given the recent development of HPV vaccines, the Department of Health should consider update current guideline to include clear instruction about HPV vaccination for sexually active people and high-risk groups. Insufficient time for recommending HPV vaccines during consultation sessions was another major barrier perceived by the participants. It is understandable as healthcare providers working in public sectors in Hong Kong are having heavy workload [40]. Developing online health promotion materials with hotline number for enquiry may be useful to tackle this barrier. After giving a clear recommendation and brief explanation during the consultation, healthcare providers may refer the attendees to access such health promotion materials for detailed information and frequently asked questions. Fact sheets and pamphlets about HPV vaccination should also be given to these attendees as reinforcements. Some concerns were related to the initiation of discussion about HPV vaccination during consultation (e.g., difficult to initiate the discussion). Skill training regarding how to initiate the discussion is hence needed. About one-third of participants perceived that recommendation of HPV vaccination would be given only if it is publicly funded. Since HPV vaccination is not included in the government’s compulsory immunization programs, it conveys the impression that the HPV vaccination is unnecessary and unimportant [41]. People in Hong Kong seem to believe that necessary vaccination should be included in the governmental scheme without any charge [41]. Such misconception should be corrected, and recommendation should be made according to attendees’ health needs. Since public STD clinics in Hong Kong are not providing HPV vaccination, relatively few participants concerned that recommendation of such vaccination would be considered as hard selling an expensive vaccine.

Perceived behavioral control is another construct that was positively associated with recommending HPV vaccination to attendees. Availability of relevant guideline/protocol and sufficient information about the HPV vaccination may be useful to increase their perceived behavioral control.

Very few of participants perceived their colleagues or supervisors would support them recommending HPV vaccination to attendees. Perceived subjective norm was not significantly associated with recommending HPV vaccination to attendees. Therefore, building up new norms supporting recommendation of HPV vaccination to attendees among healthcare providers may not be an effective approach in future health promotion. Health promotion should be evidence-based.

This study had the strengths of being theory-based and was based on a representative sample. However, it also had some limitations. First of all, recommendation of HPV vaccination to attendees was self-reported, report bias might exist. Second, we did not use items/scales validated by previous studies. Scales constructed for this study demonstrated acceptable internal reliability; however, they have not been externally validated. Third, given the limited number of healthcare providers working in public STD clinics in Hong Kong, the sample size was relatively small as compared to other similar studies [17–26]. However, since the effect size of non-significant associations (odds ratios) were quite small, the non-significance cannot hence be fully explained by the relatively small sample size. Fourth, this study did not include healthcare providers working in private STD clinics; their practices related to recommending HPV vaccination might be different from those working in public clinics. Moreover, due to the limited length of the questionnaire, most of the variables on knowledge and perceptions did not specific to female or male attendees. Therefore, we did not build different models using different variables specific to recommending HPV vaccination to female and male attendees. Furthermore, causality could not be established as this was a cross-sectional study.

## Conclusion

Sexually active people and some high-risk groups can also benefit from HPV vaccination. STD clinics are ideal settings that allow healthcare providers to access these groups and promote HPV vaccination. Only few of healthcare providers working in public STD clinics in Hong Kong had recommended HPV vaccination to their attendees. Health promotion targeting these healthcare providers is warranted. Such promotion should modify their attitudes and perceived behavioral control related to recommending HPV vaccination to attendees.

## Supporting information

S1 Survey questionnaire (DOCX)
