# Supplementary material for: Facilitators and barriers for healthcare providers to recommend HPV vaccination to attendees of public sexually transmitted diseases clinics in Hong Kong, China
Source: PLoS One. 2019 Jan 9;14(1):e0209942. doi: 10.1371/journal.pone.0209942 (PMC6326499; doi:10.1371/journal.pone.0209942)
Supplement: S1 Survey questionnaire — (DOCX) [file pone.0209942.s001.docx]

Dear colleague

You are invited to participate my study. The nature of the study is MPH thesis. My research topic is “**Knowledge and perception of HPV vaccines, and factors influencing healthcare providers’ recommendation of HPV vaccines to patients attending public sexually transmitted disease clinics in Hong Kong** “.

This self-reported questionnaire is anonymous. Your name and signature are not required. All data are kept confidential and it will be kept for 3 months upon the completion of the study and then it will be destroyed.

My study subjects are healthcare providers who are currently working in public STD clinics. Your participation is highly appreciated and useful.

Implied consent is assumed when you voluntarily return completed questionnaire. Enclosed envelops are suggested to return your completed survey.

Ethical approvals are obtained from both Ethics Committee of CUHK and Department of Health.

If you have any questions, please feel free to contact Ms Lee Ying Ying.

Thank you for your participation!!!

Socio-demographic data

1. Profession: Doctor □ Registered Nurse □ Enrolled Nurse □
2. Gender: Male □ Female □
3. Years of practice in STD clinic: <5 years□ 5-10 years□ 11-15 years□ 16-20 years□ >20 years□
4. Age: 20-30 □ 31-40 □ 41-50 □ 51-60 □ 61-70 □
5. Education level: Diploma □ Bachelor Degree □

Post-graduated Diploma/ Master’s Degree/ Above □

1. Marital status: Single □ Married □ Divorced □ Widowed □
2. Religion: Nil □ Catholic □ Christian □ Buddha □ Others:
3. Number of patients seen per week in your clinic: 0-30□ 31-99□ ≥100□

Knowledge

Based on your knowledge to HPV vaccine. Please ✓ the correct answer.

| Question |
| --- |
| 1. Is there a vaccine that protects against HPV for woman?   Yes □ No or Not Sure □ |
| 1. Is there a vaccine that protects against HPV for men?   Yes □ No or Not Sure □ |
| 1. People who are sexually active cannot receive HPV vaccine   Yes □ No □ |
| 1. People who have been diagnosed with HPV cannot be given HPV vaccine   Yes □ No □ |
| 1. People with genital warts cannot be given HPV vaccines   Yes □ No □ |
| 1. HIV-positive women cannot get HPV vaccine   Yes □ No □ |
| 1. HPV vaccination is safe for pregnant women   Yes □ No □ |

How strongly would you agree or disagree the following sentences related to immunizing your patients

against HPV? Please circle your choices.

| 1. HPV vaccine is highly effective in preventing HPV / HPV-related diseases among sexually active men and women   Strongly disagree 1 2 3 4 5 Strongly agree |
| --- |
| 1. People who have already infected with one or more HPV types can still get protection from other HYP types in the vaccines   Strongly disagree 1 2 3 4 5 Strongly agree |
| 1. For people who are infected with HPV, HPV vaccination is effective in protecting them from having genital warts or related cancers   Strongly disagree 1 2 3 4 5 Strongly agree |
| 1. HPV vaccine is highly effective in preventing HPV / HPV-related diseases among HIV positive people   Strongly disagree 1 2 3 4 5 Strongly agree |

How strongly would you agree or disagree the following sentences related to immunizing your patients

against HPV? Please circle your choices.

| 1. Recommending HPV vaccines to patients will be regarded as hard selling an expensive vaccine   Strongly disagree 1 2 3 4 5 Strongly agree |
| --- |
| 1. Time is not sufficient in each consultation session for recommending HPV vaccines to patients   Strongly disagree 1 2 3 4 5 Strongly agree |
| 1. It is difficult to initiate the conversation about HPV vaccines in the consultation   Strongly disagree 1 2 3 4 5 Strongly agree |
| 1. HPV vaccination is not related to the reasons of consultation   Strongly disagree 1 2 3 4 5 Strongly agree |
| 1. It is not my responsibility to recommend HPV vaccines to patients   Strongly disagree 1 2 3 4 5 Strongly agree |
| 1. There is lack of guideline or protocol for me to recommend HPV vaccines to patients   Strongly disagree 1 2 3 4 5 Strongly agree |
| 1. Recommendation of HPV vaccines will be given to patients only if vaccination is publicly funded   Strongly disagree 1 2 3 4 5 Strongly agree |

| 1. My **colleagues** would like me to recommend HPV vaccine to patient in the next 12 months   Strongly disagree 1 2 3 4 5 Strongly agree |
| --- |
| 1. My **supervisors** would like me to recommend HPV vaccine to patient in the next 12 months   Strongly disagree 1 2 3 4 5 Strongly agree |

| 1. I am confident I can recommend HPV vaccine to patient in the next 12 months, even if the consultation session / health education is busy   Very unconfident 1 2 3 4 5 Very confident |
| --- |
| 1. I am confident I can recommend HPV vaccine to patient in the next 12 months, if I get the information / pamphlet of HPV vaccine   Very unconfident 1 2 3 4 5 Very confident |
| 1. I am confident I can recommend HPV vaccine to patient in the next 12 months, if there is guideline / protocol from the department   Very unconfident 1 2 3 4 5 Very confident |

Practice

| 1. Have you ever recommended HPV vaccines to eligible **male** patients in the **last 12 months**?   Yes □ No □  If YES, how many patient have you recommended: <5 □ 5-10 □ >10 □ |
| --- |
| 1. Have you ever recommended HPV vaccines to eligible **female** patients in the **last 12 months**?   Yes □ No □  If YES, how many patient have you recommended: <5 □ 5-10 □ >10 □ |
